# Endovascular treatment of abdominal aortic rupture after percutaneous lithotripsy

**DOI:** 10.1590/0100-3984.2019.0125

**Published:** 2021

**Authors:** Vinicius Adami Vayego Fornazari, Rômulo Florêncio Tristão Santos, Thiago Franchi Nunes, Ricardo Abdala da Silva Oliveira, Denis Szejnfeld

**Affiliations:** 1 Sector of Interventional Radiology and Angiography, Department of Diagnostic Imaging, Universidade Federal de São Paulo (EPM-Unifesp), São Paulo, SP, Brazil.; 2 Universidade Federal de Mato Grosso do Sul (UFMS), Campo Grande, MS, Brazil.; 3 Instituto do Coração do Hospital das Clínicas da Faculdade de Medicina da Universidade de São Paulo (InCor/HC-FMUSP), São Paulo, SP, Brazil.

## INTRODUCTION

Extracorporeal shockwave lithotripsy (ESWL) is a type of urological intervention used for the treatment of stones smaller than 2 cm located in the renal pelvis or upper portion of the ureter^(1-4)^. Potential complications of this procedure include subcapsular renal hematoma, pseudoaneurysm, arteriovenous fistula, abscess formation in the psoas muscle, thrombosis of the portal or iliac vein, and-the most disastrous and potentially fatal complication-abdominal aortic aneurysm (AAA) rupture^(1,2)^.

Studies have shown that AAA is a potential risk factor for ESWL-induced arterial rupture^(1,2)^, because of calcifications of the arterial walls resulting from atherosclerotic disease, given that the mechanical energy of the shockwaves can disperse up to 12 cm from the focal point; although the intensity of the waves decreases progressively as the distance from the target increases, they can still have a deleterious effect on parietal calcifications^(4)^. Despite the scarcity of studies on this topic, we believe that advanced age and short distances between vascular parietal calcifications and renal pelvis calculi may be risk factors for AAA rupture during ESWL.

There is not sufficient scientific evidence to define the safety of ESWL in patients with AAA, a controversial subject in the literature^(1-6)^. In a review of the literature, Tse et al.^(4)^ discovered that, among the reported cases of patients with AAA who underwent ESWL, there were no vascular complications in 18, rupture of the AAA occurred in 6, and aortic rupture in a nonaneurysmal calcified aorta occurred in 2. In the present article, we will demonstrate the endovascular correction of abdominal aorta rupture in a patient submitted to ESWL.

## PROCEDURE

Symptoms of AAA rupture may include severe low back pain, general malaise, nausea, vomiting, dizziness, and blurred vision. Physical examination may show pallor, hypotension, tachycardia, and a pulsatile abdominal mass. In cases of hemodynamic instability, the patient must first be stabilized, and, when possible, should undergo computed tomography (CT) angiography of the abdomen, which is the imaging modality of choice for the diagnosis and planning of endovascular treatment. Rupture of an AAA is more likely to occur in the retroperitoneal space than in the peritoneal cavity, mortality rates being higher if it occurs in the latter^(4)^.

**Figure 1:**
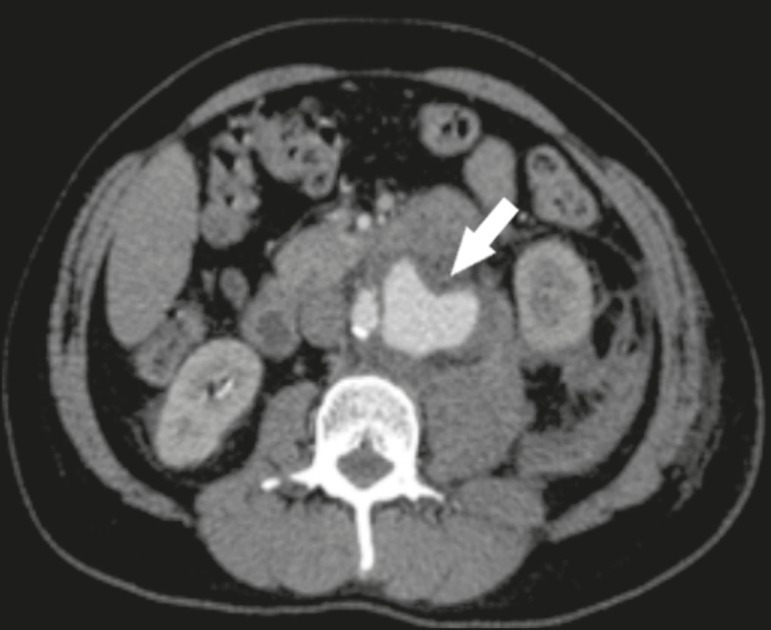
Axial CT angiography of the abdomen with iodinated contrast, in the arterial phase, showing a pseudoaneurysm to the left of the infrarenal abdominal aorta, with retroperitoneal extravasation due to restriction to the psoas muscle group (arrow).

**Figure 2:**
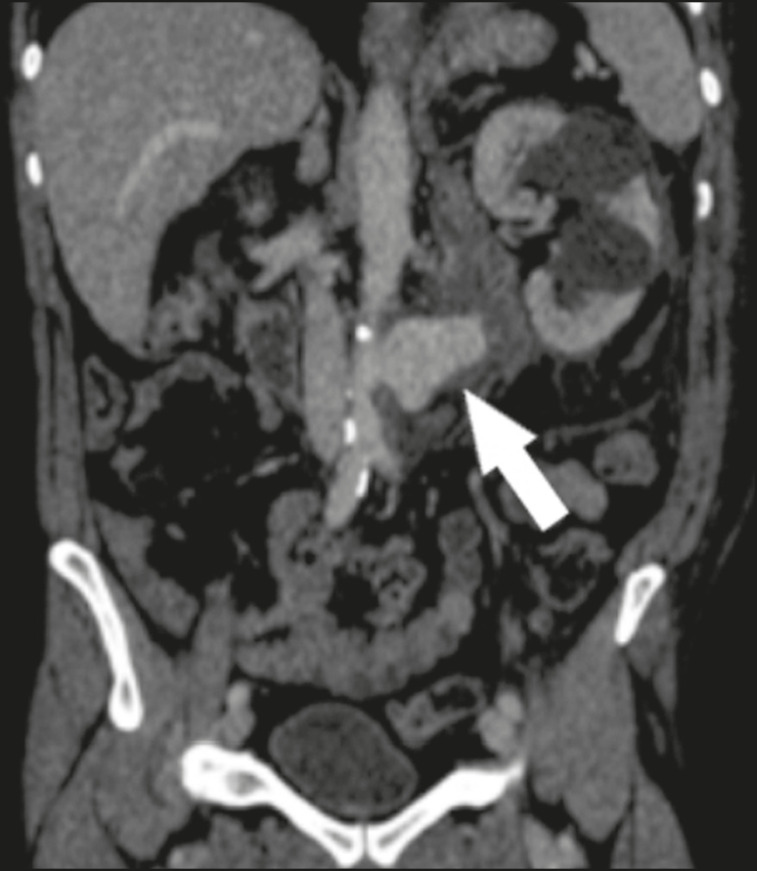
Coronal CT angiography of the abdomen with iodinated contrast, in the arterial phase, showing pseudoaneurysm to the left of the infrarenal abdominal aorta (arrow).

When diagnosing AAA rupture by CT angiography (Figures 1 and 2), clinical patient stabilization measures must be taken together and despite a rapid endovascular approach, CT angiography has greater therapeutic potential than does conventional surgery because the former has a shorter execution time, less morbidity, and low technical complexity^(7)^.

**Figure 3:**
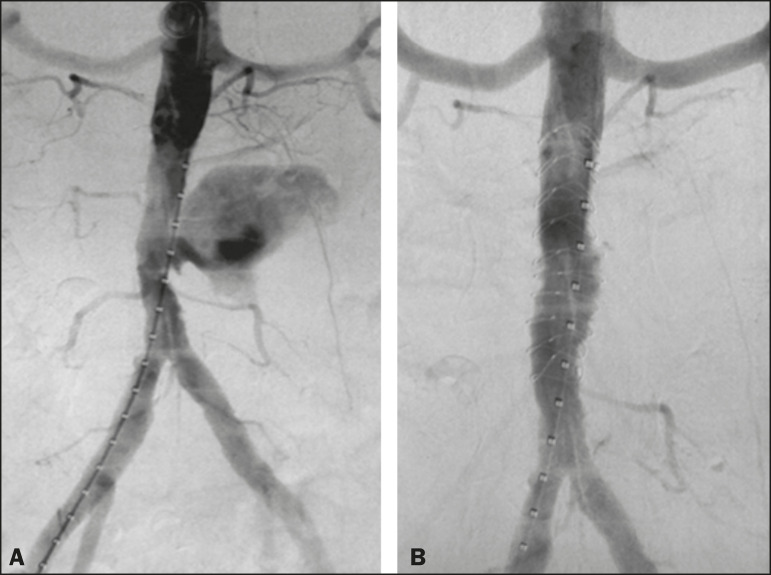
A: Aortic arteriography showing extravasation of contrast media, together with a pseudoaneurysm. B: Aortic arteriography after endovascular repair of the pseudoaneurysm.

The endovascular approach begins in the hemodynamics room, with the patient in the supine position. Ultrasound-guided puncture of the right common femoral artery, by the Seldinger technique, is followed by insertion of a 6 Fr femoral introducer, a stiff guidewire, and a pigtail catheter for aortic angiography, aiming at planning the procedure, which will be involve coverage of the rupture site (pseudoaneurysm) with a coated stent (Figure 3A), through the use of a roadmap. A follow-up CT angiography is necessary in order to verify the proper positioning of the stent and that there is no extravasation of blood (Figure 3B). The type of post-procedure clinical support (semi-intensive or intensive) depends on the clinical status of the patient at admission rather than on the endovascular treatment in question.

Follow-up evaluation can be performed after one week at an outpatient clinic, and a follow-up CT angiography can be performed after 30 days. Patients should be reevaluated at 12 months after the procedure, with Doppler ultrasound evaluation of the abdominal aorta, iliac arteries, renal arteries, and superior mesenteric artery.
